# Human Neonatal Thymus Mesenchymal Stem/Stromal Cells and Chronic Right Ventricle Pressure Overload

**DOI:** 10.3390/bioengineering6010015

**Published:** 2019-02-09

**Authors:** Josue Chery, Shan Huang, Lianghui Gong, Shuyun Wang, Zhize Yuan, Joshua Wong, Jeffrey Lee, Sean Johnson, Ming-Sing Si

**Affiliations:** 1 Department of Cardiac Surgery, University of Michigan, Ann Arbor, MI 48109, USA; jchery03@yahoo.com (J.C.); huangshancomeon@yeah.net (S.H.); lhgong@umich.edu (L.G.); wangshuy@umich.edu (S.W.); yuanzhize0402@126.com (Z.Y.); Joshua.wong@advocatehealth.com (J.W.); leejeffr@umich.edu (J.L.); seanlj@umich.edu (S.J.); 2 Central South University, Changsha 41000, China

**Keywords:** right ventricle, pressure overload, heart failure, angiogenesis, mesenchymal stem cells, antioxidant, extracellular type superoxide dismutase

## Abstract

Right ventricle (RV) failure secondary to pressure overload is associated with a loss of myocardial capillary density and an increase in oxidative stress. We have previously found that human neonatal thymus mesenchymal stem cells (ntMSCs) promote neovascularization, but the ability of ntMSCs to express the antioxidant extracellular superoxide dismutase (SOD3) is unknown. We hypothesized that ntMSCs express and secrete SOD3 as well as improve survival in the setting of chronic pressure overload. To evaluate this hypothesis, we compared SOD3 expression in ntMSCs to donor-matched bone-derived MSCs and evaluated the effect of ntMSCs in a rat RV pressure overload model induced by pulmonary artery banding (PAB). The primary outcome was survival, and secondary measures were an echocardiographic assessment of RV size and function as well as histological studies of the RV. We found that ntMSCs expressed SOD3 to a greater degree as compared to bone-derived MSCs. In the PAB model, all ntMSC-treated animals survived to the study endpoint whereas control animals had significantly decreased survival. Treatment animals had significantly less RV fibrosis and increased RV capillary density as compared to controls. We conclude that human ntMSCs demonstrate a therapeutic effect in a model of chronic RV pressure overload, which may in part be due to their antioxidative, antifibrotic, and proangiogenic effects. Given their readily available source, human ntMSCs may be a candidate cell therapy for individuals with congenital heart disease and a pressure-overloaded RV.

## 1. Introduction

Pressure overload has a profound and deleterious effect on the right ventricle (RV) [1]. With pressure overload, the RV wall stress increases, which results in an accumulation of muscle mass leading to increased RV wall thickness. Chronic pressure overload induces a maladaptive remodeling response, which progresses to decompensation and RV failure [1]. Conversely, in response to pressure overload, the left ventricle (LV) undergoes compensatory cardiomyocyte hypertrophy with increased angiogenesis and the antioxidant enzyme extracellular superoxide dismutase (SOD3) [2], thereby diminishing the deleterious effects of the pressure overload [1].

Right ventricle pressure overload is encountered in many congenital heart defects [3]. On a histological level, the RV experiences loss of myocardial capillary density as well as mitochondrial dysfunction on the cellular level, which occurs secondary to oxidative stress [4]. Conceivably, the pathophysiology of RV dysfunction secondary to pressure overload can be mitigated if capillary density is preserved and oxidative stress is reduced.

Perivascular mesenchymal stem/stromal cells (MSCs) actively secrete cytokines, extracellular matrix, growth factors, and immunomodulators that enable them to have a multitude of paracrine and juxtacrine effects on their local environment [5]. We previously found that human neonatal thymus mesenchymal stem cells (ntMSCs) isolated from discarded tissue obtained from cardiac surgical procedures promote angiogenesis and preserve cardiac function after ischemia [6,7].

SOD3 is highly expressed in the heart and the vascular smooth muscle cells of the aorta [8]. Furthermore, human bone marrow-derived MSCs exert their antioxidant properties in part via the secretion of the extracellular superoxide dismutase (SOD3) [9]. However, the ability of ntMSCs to express the antioxidant SOD3 is unknown. In this study, we evaluated our hypothesis that ntMSCs could express and secrete SOD3 and improve RV function and survival in the setting of chronic pressure overload in vivo.

## 2. Materials and Methods

Cells and Cell Culture: All studies were performed under an approved protocol from the University of Michigan Institutional Review Board. The isolation, detailed characterization, and culture of neonatal bone (nb)MSC and ntMSC lines used in this study have been described previously [7,10]. Briefly, after informed consent was given by the parents, discarded thymus and sternal bone tissue from infant heart operations were mechanically minced into <3 mm fragments under sterile conditions. Tissue fragments were placed in 100 mm culture dishes and submerged in MSC media (Dulbecco’s modified Eagle medium, with high glucose concentration, GLUTAMAX I, 10% heat-inactivated fetal bovine serum, 100 U/mL penicillin, and 100 μg/mL streptomycin, all from Gibco, Carlsbad, CA). Tissue fragments were incubated for 10–14 days prior to removal. MSCs that had migrated from the tissue explants were allowed to achieve 80% confluence prior to passaging with trypsin/EDTA (Gibco). Unrelated adult bone marrow (ab)MSCs were obtained from Lonza (Basel, Switzerland) and ATCC (Manassas, VA, USA). Human umbilical vein endothelial cells (HUVECs) were cultured in EGM-2 (both from Lonza) with growth factors under standard conditions, unless stated otherwise. Unless specified otherwise, all experiments were performed with cells from passages 3–9.

SOD3 Expression: SOD3 expression was confirmed in ntMSCs with Western blot. Protein was isolated from ntMSCs cultured under standard conditions and then quantified by bicinchoninic acid (BCA) assay (Pierce, Rockford, IL, USA). Next, 25 μg of total protein was loaded onto SDS-polyacrylamide gel electrophoresis and transferred to nitrocellulose membranes. Membranes were blocked in 5% skimmed milk for 1 h at room temperature and then incubated with primary antibody (Monoclonal Mouse IgG1 anti-Human SOD3/EC-SOD Antibody, 1:500 dilution, Clone # 713707, Catalog Number: MAB34201, R&D Systems, Minneapolis, MN, USA) overnight at 4 °C. Goat anti-mouse IgG (H+L) antibody IRDye 680 conjugated was applied for 1 h at room temperature (1∶10,000, Rockland Immunochemicals, Inc., Limerick, PA, USA). Visualization and quantification were carried out with the LI-COR Odyssey® scanner and software (LI-COR Biosciences, Lincoln, NE, USA).

Differential SOD3 transcript expression in the different cell types and culture conditions were determined with qPCR. Total RNA was extracted from cells or cell sheets using the RNeasy Mini Kit (Qiagen, Valencia, CA, USA). Reverse transcription was carried out using the High-Capacity cDNA Reverse Transcription kit with random primers (Applied Biosystems, Foster City, CA, USA). Quantitative real-time polymerase chain reaction was performed in StepOne Plus Real-time PCR system (Applied Biosystems) with a reaction mixture (10 μL) containing cDNA, forward and reverse primers (see below), and 1× iTaq Universal SYBR Green Supermix (Bio-Rad Laboratories, Hercules, CA, USA). The expression of each gene was normalized to the expression of β-actin. Mean cycle threshold (Ct) value was calculated as the average of triplicates for each gene, and the fold change in gene expression was calculated based on 2-ΔΔCT method. SOD3 qPCR primers used in this study are listed here: Fwd: 5’- CTGGGTGCAGCTCTCTTTTC-3’ and Rev: 5’-ACATGTCTCGGATCCACTCC-3’.

Scaffoldless ntMSC Cell Sheet Generation: Scaffoldless ntMSC cell sheets were generated by culturing 4 × 10^6^ ntMSCs in thermoresponsive polymer-coated 35-mm culture dishes (Nunc™ Dishes with UpCell™ Surface, Thermo Fisher Scientific, Waltham, MA, USA) for 48 h under standard culture conditions. These cell sheets do not contain any exogenously-added scaffold or hydrogel. After exposing the dishes to room temperature for 10 min, cell sheets lifted off the culture surface and were harvested for use or measurement of SOD3 expression.

Right Ventricle Pressure Overload Model: All animal procedures were performed under institutional approval. RNU nude rats (200–225 g) were used for this study, and all animals received a main pulmonary artery (PA) band at an initial operation. Animals that survived this procedure and had a PA band peak gradient of 25–60 mmHg at one week were included in the study and underwent a redo left thoracotomy two weeks post PA band placement. During the second procedure, the control group did not receive treatment (*n* = 7) whereas the experimental group (*n* = 8) received ntMSC cell sheet application to the free wall of the RV. Animals were followed out to the study endpoint of 100 days. During the study, it was decided to permit the treatment group to continue to approximately 1 year because all animals in this group had survived to the original study endpoint.

For both procedures, induction was initiated with inhalational isofluorane (3%) mixed with 1 L/min of oxygen followed by intra-peritoneal injection of ketamine/xylazine (40/5 mg/kg). Endotracheal intubation with an 18-gauge angiocatheter was then performed. Animals were then maintained on a rodent mechanical ventilator with oxygen and isoflurane delivered via endotracheal tube. A left anterolateral thoracotomy was performed, and entry into the left chest was at the third intercostal space. The PA was separated from the aorta. A custom right-angle dissector was used to pass a 7–0 Prolene suture (Ethicon Inc., Somerville, NJ, USA) around the PA, which was then quickly tied around an 18-gauge hypodermic tube. The hypodermic tube was then removed and the chest closed in standard fashion. Once spontaneously breathing, the animals were extubated and placed in a heated cage for observation until ambulatory.

Echocardiogram: Echocardiograms were performed seven days post PA band placement and just prior to euthanasia. All echocardiograms were performed under isoflurane inhalational anesthesia in a supine and left lateral position. Two-dimensional, M-mode, Doppler and tissue Doppler echocardiographic images were recorded using a Visual Sonics’ Vevo 2100 high-resolution in vivo micro-imaging system. The PA band gradient and RV wall thickness and size were assessed.

Histology and Fibrosis: After the last echocardiogram, the animals were euthanized, and the hearts were harvested after the administration of a potassium-rich solution to ensure diastolic arrest. Heart tissue was then fixed in formalin, paraffin-embedded, sectioned at 5 µm thickness, and stained with H&E and Masson’s Trichrome. Quantification of fibrosis from digitized Masson Trichrome images was analyzed using Image Pro software (Media Cybernetics, Rockville, MD, USA).

CD31+ Vascular Density: Heart sections were deparaffinized and rehydrated in gradients of ethanol. After antigen retrieval, slides were blocked in 10% donkey serum for 1 h at room temperature, then incubated with primary antibodies (goat anti-mouse/rat CD31 antibody, AF3628, R&D system, at 5 μg/mL) overnight at 4 °C. Following three washes with PBS, the sections were incubated with donkey anti-goat AF488 (Invitrogen, Eugene, OR, USA) for 1 h in dark and counterstained with DAPI and then mounted in ProLong diamond mounting medium (Molecular Probes, Eugene, OR, USA). Fluorescence images were acquired using a confocal microscope (Nikon A1, Nikon Instruments, Inc., Melville, NY, USA).

Statistical Analysis: Statistical analysis was performed using Prism 8 (GraphPad Software, San Diego, CA, USA) and IBM SPSS Version 25 (IBM Corporation, Armonk, NY, USA). The mean ± standard deviation were calculated for group data, and the Student’s *t*-test or one-way ANOVA, with or without the Tukey’s multiple comparison test, was used to compare means between control and treatment groups when appropriate. When matched ntMSCs were compared to nbMSCs, a paired *t*-test was used to compare the groups. A *p*-value less than 0.05 was deemed to be significant.

## 3. Results

### 3.1. SOD3 Expression in Different Cell Types under Various Culture Conditions

**Figure 1:**
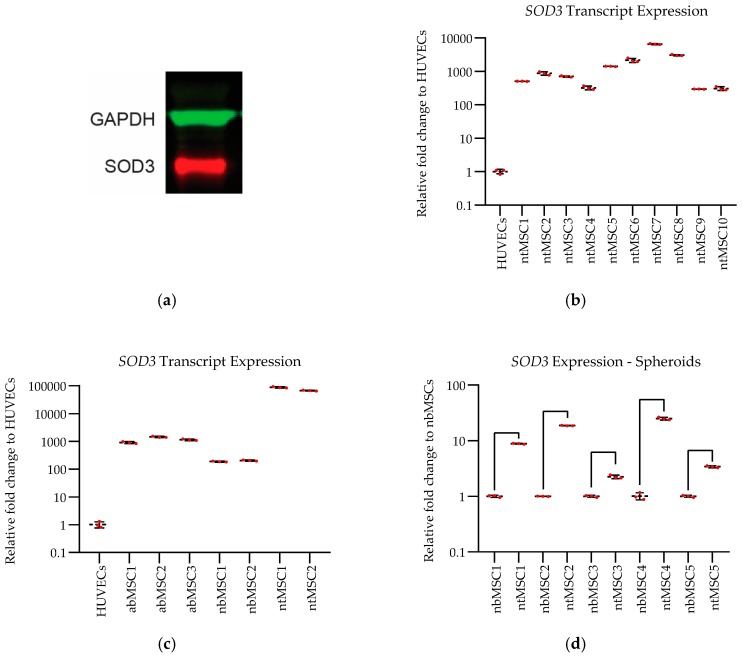

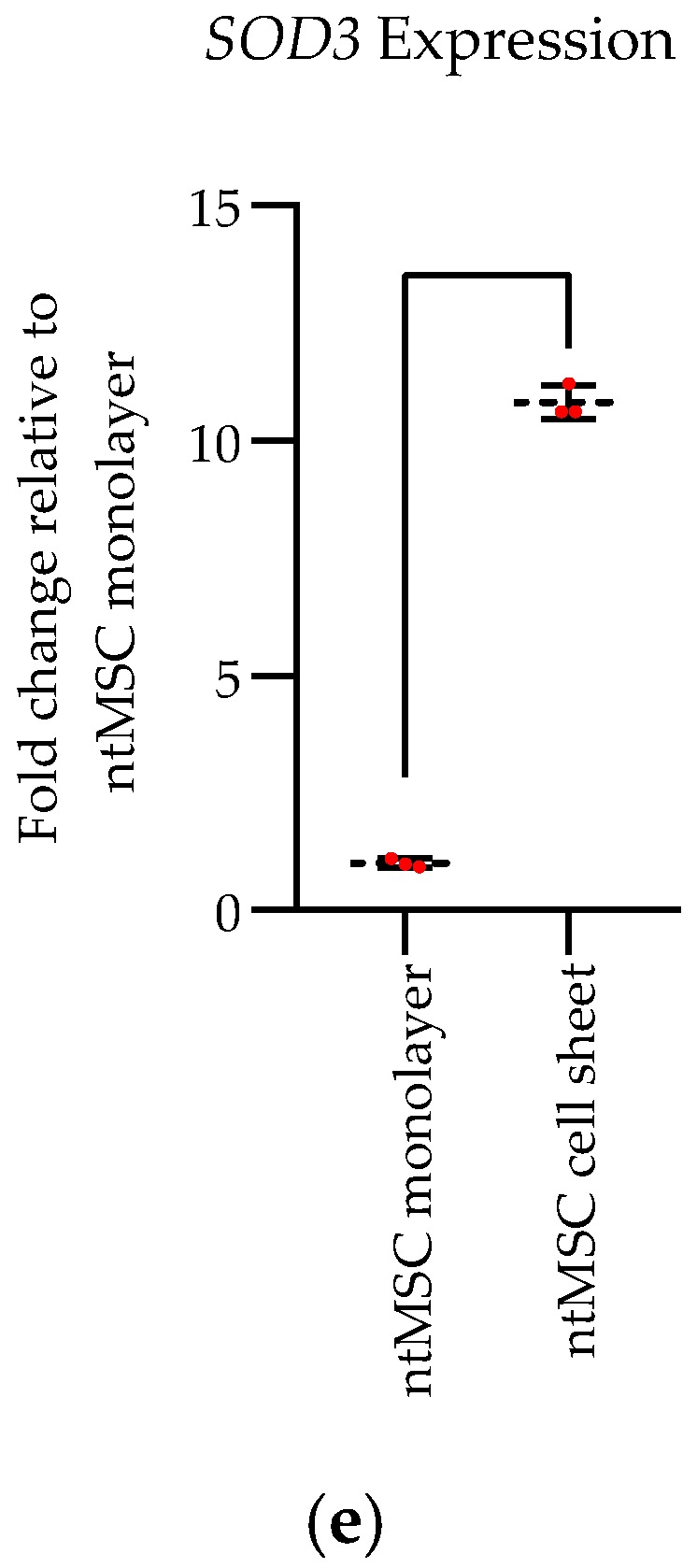
(**a**) Extracellular superoxide dismutase (SOD3) protein expressed in neonatal thymus mesenchymal stem cells (ntMSCs) cultured as monolayers; (**b**) SOD3 transcript expression in ntMSCs isolated from ten subjects were all significantly more than compared to Human umbilical vein endothelial cells (HUVECs) by one-way ANOVA (*p* < 0.0001); (**c**) SOD3 transcript expression was significantly greater in ntMSCs as compared to HUVECs, adult bone marrow (ab)MSCs, or matched neonatal bone (nb)MSCs by one-way ANOVA with the Tukey’s multiple comparison test (*p* < 0.0001); (**d**) Differential SOD3 expression was maintained in 3D spheroid culture (*p* < 0.0001); (**d**) Culture of ntMSCs in scaffoldless cell sheet form activated SOD3 transcription (*p* < 0.0001).

SOD3 protein expression was confirmed in monolayer-cultured ntMSCs by Western blot (Figure 1). Next, we determined the differential transcript expression of SOD3 in ntMSCs as compared to other cells in different culture conditions by qPCR. We observed the transcript expression of SOD3 to be several orders of magnitude greater in ntMSCs as compared to HUVECs (*p* < 0.0001) (Figure 1). Furthermore, in separate experiments, we found that ntMSCs also had a significantly greater SOD3 transcript expression as compared to HUVECs, unrelated abMSCs, and donor-matched nbMSCs (*p* < 0.0001) (Figure 1). Next, we evaluated if this difference in SOD3 transcript expression between ntMSCs and nbMSCs would persist during 3D culture. We found that ntMSC SOD3 transcription levels remained significantly greater than in nbMSCs when both were cultured in spheroids (*p* < 0.0001) (Figure 1). Furthermore, we also found that culturing ntMSCs in cell sheet form also stimulated the expression of SOD3 as compared to monolayer culture, which aligns with our prior findings that cell sheet culture of ntMSCs stimulated angiogenic gene expression (*p* < 0.0001) (Figure 1) [6]. Collectively, these results indicate that ntMSCs possess a high transcript expression of SOD3 as compared to HUVECs and other types of MSCs, and that cell sheet culture promotes further activation of SOD3 transcription.

### 3.2. Survival after PA Banding

**Figure 2:**
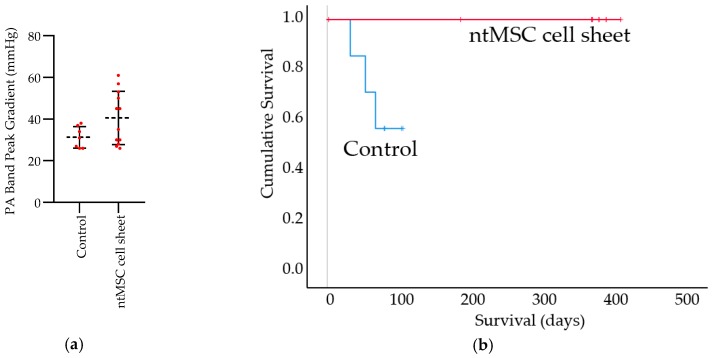
Treatment with ntMSC cell sheets improved survival after PA banding. (**a**) Control and ntMSC cell sheet treatment animals had equivalent initial PA band gradients; (**b**) Survival was significantly increased in the ntMSC cell sheet treatment group, *p* = 0.018 by log rank test.

RNU rats underwent echocardiograms 7 days after PA banding was performed and peak gradients were measured across the PA band. Animals (*n* = 1 in the control group and *n* = 2 in the treatment group) were excluded from the study because their initial PA band gradients were outside of the inclusion range. Non-treated and ntMSC cell sheet-treated PA banded rats that were included in the study did not have significantly different PA band gradients (*p* = 0.085, Figure 2).

Control animals suffered from significant cardiac-related mortality up to the 100-day endpoint (Figure 2). Control animals that suddenly died (*n* = 3) were all found to have significant pleural effusions and ascites at necropsy, and thus these deaths were adjudicated to be cardiac-related (Figure 2). In contradistinction, ntMSC cell sheet treated animals had no cardiac-related deaths in the first 100 days (one animal had to be euthanized earlier because of an unrelated foot infection and another due to a premature extubation after surgery) as well as to 1 year. The ntMSC cell sheet treatment group had a significantly different survival as compared to the control group (*p* = 0.018 by Log rank test) (Figure 2). None of the treated animals were noted to have pleural effusions or ascites at the time of euthanasia.

### 3.3. Late Echocardiogram Studies

**Figure 3:**
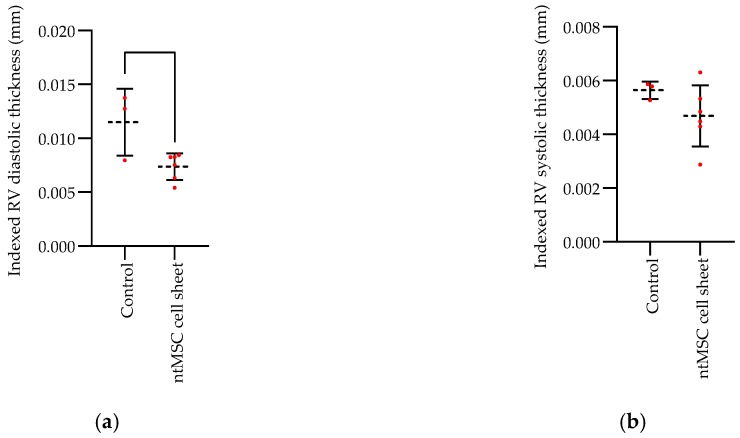

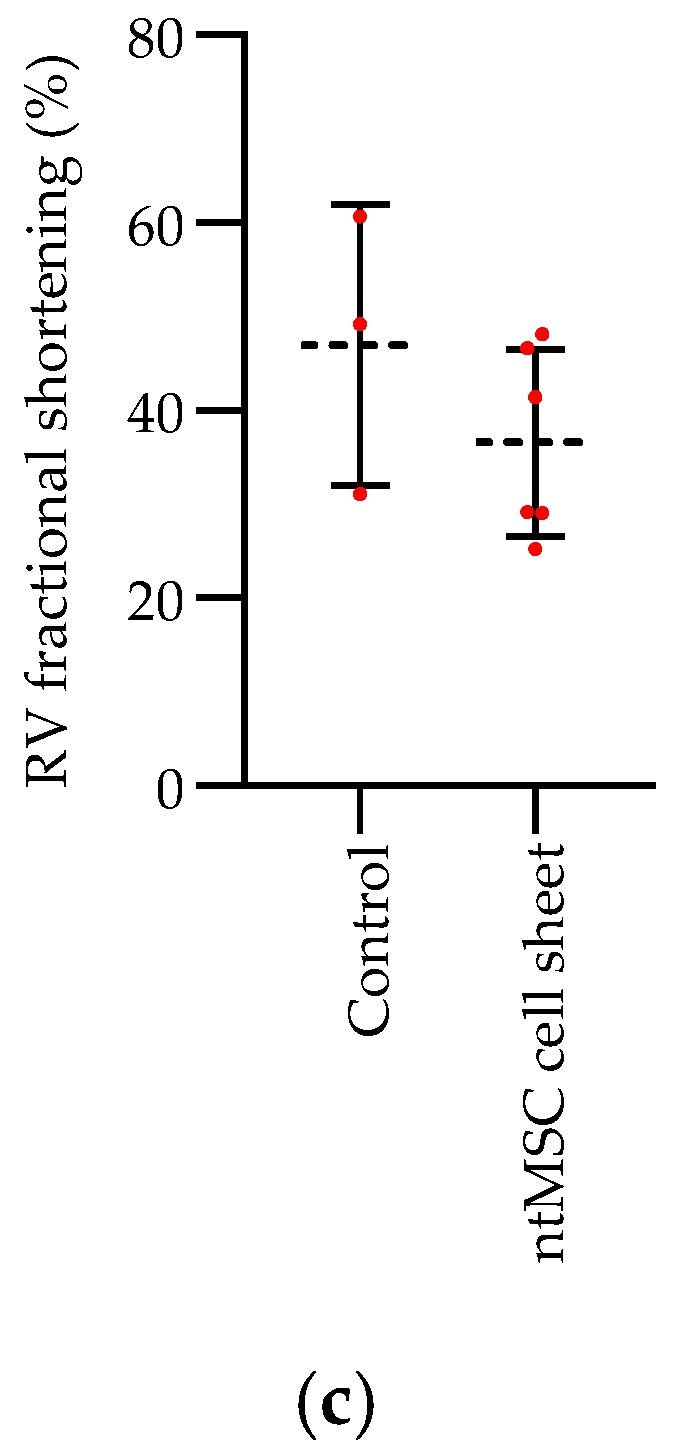
Echocardiogram studies on surviving animals (*n* = 3 controls at 100 days vs. *n* = 6 treatment animals at 1 year). (**a**) Indexed right ventricle (RV) wall thickness at diastole was significantly greater in the control group (*p* = 0.02); (**b**) Indexed RV wall thickness at systole was similar between the two groups; (**c**) RV fractional shortening was similar between the two groups.

Late echocardiogram studies were performed just prior to euthanasia for both control (*n* = 3 at 100 days) and treatment (*n* = 6 at approximately 1 year) animals. Animals that survived in the control group up to 100 days had significantly increased RV diastolic dimensions (indexed to body mass) as compared to those in the treatment group that survived to 1 year (Figure 3). Indexed RV systolic wall thicknesses were similar between the two groups (Figure 3) Neither did the extent of RV fractional shortening differ between the surviving control and treatment animals prior to euthanasia (Figure 3).

### 3.4. Histological Findings

**Figure 4:**
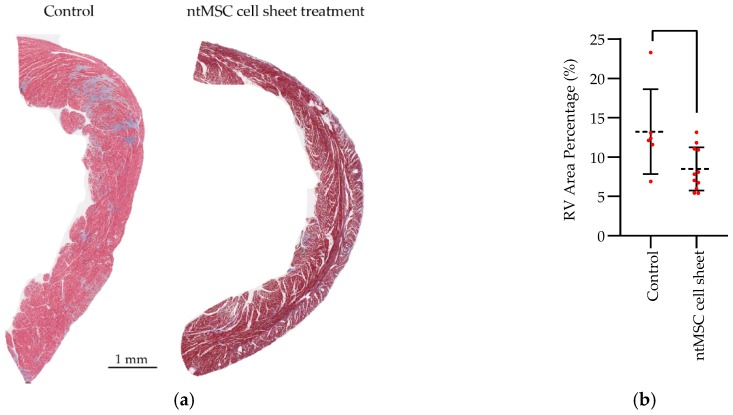

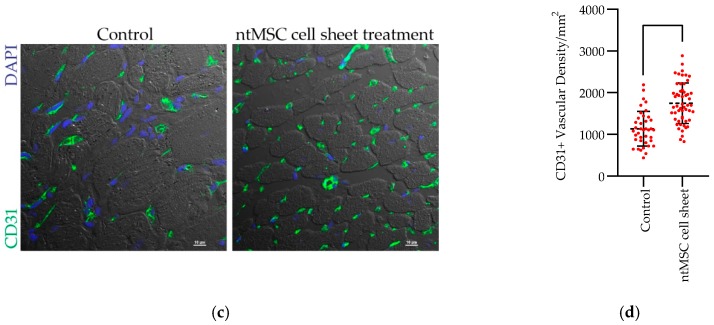
Histological studies of surviving animals. (**a**) Masson Trichrome stain of RV free wall of control and ntMSC cell sheet treatment animals (Magnification: 4× and scale bar = 1 mm); (**b**) Quantification of fibrosis area as a percentage of total RV free wall area. Fibrosis was significantly decreased in ntMSC cell sheet treatment animals (*p* = 0.03); (**c**) Immunohistochemical staining for CD31+ vascular structures in control and ntMSC cell sheet treatment RVs (Magnification: 200× and scale bar = 10 μm); (**d**) Quantification of CD31+ vascular density in the RVs from control and ntMSC cell sheet treatment animals. Treatment animals had significantly increased CD31+ vascular density as compared to controls (*p* < 0.0001).

Fibrosis is an important component of adverse remodeling in the pressure-overloaded ventricle [11]. To determine if ntMSCs could influence the formation of fibrosis in the pressure-overloaded RV, we performed Masson Trichrome staining of RVs from the PA-banded rats that survived the study (*n* = 3 controls at 100 days and *n* = 6 at 1 year) (Figure 4). Control animals had a significant degree of perivascular and interstitial fibrosis, whereas treated animals had mild perivascular fibrosis and minimal interstitial fibrosis after quantification (Figure 4).

Because capillary rarefaction is regarded to be important in the pathophysiology of heart failure induced by pressure overload [12,13] and that ntMSCs are known to have proangiogenic effects [6,7], we evaluated CD31 capillary density in the RV free wall of control and treated PA-banded animals (Figure 4). We found that CD31+ vascular density was lower in the untreated controls and significantly higher in ntMSC cell sheet-treated animals (Figure 4).

## 4. Discussion

RV failure secondary to chronic pressure overload is a complex condition encountered in congenital heart disease and pulmonary hypertension [13]. The molecular mechanisms of this important clinical condition are only partially understood. Mitochondrial dysfunction secondary to oxidative stress, capillary rarefaction, and fibrosis contribute to RV failure induced by pressure overload [4,13]. We demonstrated that ntMSCs can mitigate the deleterious effect of pressure overload on the RV, and that this beneficial effect may occur by several potential mechanisms. First, ntMSCs can promote angiogenesis through the secretion of several proangiogenic factors. Second, ntMSCs may be an important source of SOD3 in the pressure-overloaded RV that is prone to cellular injury from oxidative stress. However, the significance of each of these effects demonstrated to be present in vitro to the overall preservation of RV function in vivo is unknown. Thus, future work will need to be performed to examine each of these mechanisms individually.

The optimal delivery method of MSCs to the injured heart remains unknown. Here we show that ntMSCs in cell sheet form may increase the therapeutic potency of these cells. Furthermore, the delivery of a large number of these cells (4 × 10^6^) was made possible by using this method, and this would not have been possible using an intramyocardial injection approach. Since the prevailing thought is that MSCs and other stem cells improve cardiac function via a paracrine mechanism [11,14], increasing the number of MSCs could result in an increased paracrine and therapeutic effect.

Like hypoplastic left heart syndrome, single ventricle heart disease leaves the RV susceptible to heart failure from chronic pressure overload [3,12]. Staged palliative surgeries, culminating in the Fontan procedure, whereby the arterial and venous circulations are connected in series, remain the mode of treatment for these patients [15]. At the first stage, during the Norwood procedure, part of the thymus is resected at the time of the sternotomy for improved exposure. Here, we demonstrated the feasibility of harvesting MSCs from this discarded thymus tissue and also demonstrated their beneficial effects in a relevant animal model of chronic RV pressure overload. Therefore, MSCs could be isolated from discarded thymus tissue of these patients during the neonatal period, cryopreserved, and utilized later in life for therapeutic purposes when these patients eventually develop RV failure.

The seminal ELPIS trial (Allogeneic Human MEsenchymal Stem Cell Injection in Patients with Hypoplastic Left Heart Syndrome: An Open Label Pilot Study) is being initiated to evaluate the safety and efficacy of allogeneic bone marrow derived MSCs in HLHS patients [16]. While this trial represents a significant advance for cardiac regenerative therapies for patients with congenital heart disease, the potency of stem cell therapy for cardiac disease in adults remains under scrutiny [17]. Our findings suggest relevant biological activity of ntMSCs to preserve RV function in the setting of pressure overload. Therefore, the therapeutic potential and potency of MSCs derived from the neonatal thymus and from other tissue sources need to be evaluated head-to-head in an unbiased fashion to determine the best and most potent candidate for therapy.
